# Hole-limited electrochemical doping in conjugated polymers

**DOI:** 10.1038/s41563-023-01601-5

**Published:** 2023-07-06

**Authors:** Scott T. Keene, Joonatan E. M. Laulainen, Raj Pandya, Maximilian Moser, Christoph Schnedermann, Paul A. Midgley, Iain McCulloch, Akshay Rao, George G. Malliaras

**Affiliations:** 1 Electrical Engineering Division, Department of Engineering, University of Cambridge, Cambridge, UK; 2 Cavendish Laboratory, University of Cambridge, Cambridge, UK; 3 Department of Materials Science and Metallurgy, University of Cambridge, Cambridge, UK; 4 Laboratoire Kastler Brossel, École Normale Supérieure, Université PSL, CNRS, Sorbonne Université, Collège de France, Paris, France; 5 Department of Chemistry, University of Oxford, Oxford, UK; 6 KAUST Solar Center, King Abdullah University of Science and Technology, Thuwal, Saudi Arabia

**Keywords:** Electronic devices, Characterization and analytical techniques, Electronic properties and materials, Electrochemistry

## Abstract

Simultaneous transport and coupling of ionic and electronic charges is fundamental to electrochemical devices used in energy storage and conversion, neuromorphic computing and bioelectronics. While the mixed conductors enabling these technologies are widely used, the dynamic relationship between ionic and electronic transport is generally poorly understood, hindering the rational design of new materials. In semiconducting electrodes, electrochemical doping is assumed to be limited by motion of ions due to their large mass compared to electrons and/or holes. Here, we show that this basic assumption does not hold for conjugated polymer electrodes. Using operando optical microscopy, we reveal that electrochemical doping speeds in a state-of-the-art polythiophene can be limited by poor hole transport at low doping levels, leading to substantially slower switching speeds than expected. We show that the timescale of hole-limited doping can be controlled by the degree of microstructural heterogeneity, enabling the design of conjugated polymers with improved electrochemical performance.

## Abstract (web summary)

Electrochemical doping is assumed to be limited by ion motion due to large mass in mixed ionic-electronic conductors. Here, the authors reveal in a typical polythiophene that electrochemical doping speeds are limited by poor hole transport at low doping levels, leading to much slower switching speeds than expected.

## Main

Organic mixed ionic-electronic conductors (OMIECs) have emerged for applications in bioelectronics where their properties can be exploited for sensing^1^, neural recording^2^, complementary logic^3^, energy harvesting^4^ and storage^5^, and memory^6^. These devices benefit from a combination of excellent electronic properties (hole mobilities of 1 to 10 cm^2^ V^−1^ s^−1^) and high ionic conductivities (up to 10^−2^ S cm^−1^)^7^. At the core of OMIEC device operation is the conversion of ionic currents from an external electrolyte to modulations in the electronic carrier density of a conjugated polymer (CP)^8^.

The performance of OMIECs for use in organic electrochemical transistors (OECTs) is benchmarked by their ability to amplify small voltage signals. The magnitude of amplification results primarily from the electronic properties of the material, namely the hole and/or electron mobility, *µ*, and the amount of charge modulation or (de)doping for a given change in potential (called volumetric capacitance, C^*^), where the product µC^*^ is the typical materials figure of merit^7^. However, this quantity does not capture critical device properties such as speed of (de)doping, which is presumed to primarily depend on the ionic transport properties^9^ since ionic masses greatly exceed those of electronic carriers.

**Fig. 1:**
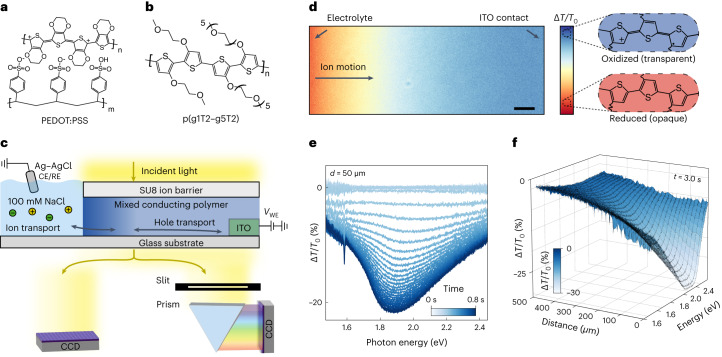
Operando transmission microscopy to monitor electrochemical doping in mixed conducting polymers. **a**,**b**, Chemical structures of PEDOT:PSS (**a**) and p(g1T2-g5T2) (**b**). **c**, Experimental geometry where ions are injected and/or extracted from the left and electrons are extracted and/or injected from the right (note the Ag–AgCl electrode is not drawn to scale). The transmitted 2D image is either directly imaged in greyscale on a CCD or passed through a 1D slit and prism to obtain a 1D-spatial 1D-spectral image. **d**, Differential transmission intensity (∆*T*/*T*_0_) image. Scale bar, 50 μm. **e**, Time-resolved ∆*T*/*T*_0_ spectra at a distance *d* = 50 µm from the polymer–electrolyte interface. **f**, ∆*T*/*T*_0_ spectra as a function of distance for a single time point *t* = 3.0 s after applying *V*_WE,f_ during electrochemical dedoping of PEDOT:PSS (*V*_WE,i_ = 0 V to *V*_WE,f_ = 0.8 V versus Ag–AgCl). The decrease in transmitted intensity corresponds to reduction of the thiophene backbone.

The most widely used OMIEC is PEDOT:PSS (Fig. 1a), which is a blend of the semiconducting polymer PEDOT, which gives the composite its electronic properties, and an ion conducting polymer, PSS, which also acts as a dopant. While PEDOT:PSS is the standard benchmark for OMIEC performance (µC^*^ of roughly 50 F cm^−1^ V^−1^ s^−1^), there has been a recent trend in developing higher performance materials by modifying traditional semiconducting polymer backbones via addition of hydrophilic sidechains^10^. This approach has led to the high performing polymer p(g1T2-g5T2) (Fig. 1b). The homopolymer p(g1T2-g5T2) is an intrinsic semiconductor with a polythiophene backbone and hydrophilic ethylene glycol sidechains to enable ion transport, where the distribution of sidechains between adjacent bithiophene units is optimized to maximize performance (µC^*^ of 500 F cm^−1^ V^−1^ s^−1^)^11^. While newly developed materials have been optimized for electronic transport, little is known about their ionic transport properties.

Like other mixed ionic-electronic conductors including lead-halide perovskites^12^ and battery electrodes^13^, it is critical to understand the interaction between and transport properties of ionic and electronic charges in OMIECs. However, achieving a mechanistic picture of mixed transport is challenging due to the dynamic interactions among ions, holes and/or electrons and the material microstructure. Furthermore, it is not possible to probe the transport of each carrier type independently with standard electrical and electrochemical techniques. The lack of mechanistic understanding in turn holds back the rational design of new mixed conducting materials with optimized properties^14^. Hence, measurements that differentiate between ionic and electronic transport are needed to improve the fundamental understanding of mixed conducting materials.

Here, we leverage the electrochromic response of CPs to study their mixed ionic-electronic transport properties using operando optical microscopy. We explore doping dynamics in the most widely used OMIEC PEDOT:PSS and compare its performance to the current champion material system p(g1T2-g5T2). The results show that ion motion does not necessarily limit the rate of doping in OMIECs. Instead, at low hole concentrations, heterogeneity in the hole energetic landscape can result in lower hole drift compared to ions, giving rise to localized hole-limited electrochemical doping. Hole-limited doping has consequential effects on critical device parameters including switching speed that, to the best of our knowledge, is not captured by any existing models describing the kinetics of electrochemical doping in CPs.

## Optical measurements of ion and hole dynamics

We use a ‘moving front’ device architecture^15^, as shown in Fig. 1c, which allows for monitoring of lateral ion motion through a 500 µm-long OMIEC film during electrochemical (de)doping. The device consists of an SU8 ion barrier covering the OMIEC film such that the end opposite the indium-tin oxide (ITO) contact is exposed to the aqueous 0.1 M NaCl electrolyte. This geometry ensures that ions and holes are injected into or extracted from the OMIEC at opposite ends of the film. In the orthogonal direction, a focused white light (520 to 780 nm, Supplementary Fig. 1) is used to image the transmissivity, and therefore oxidation state of the CP, giving a snapshot of the distribution of holes at any point in time (Fig. 1d). The changes in transmissivity correspond to large changes in bulk carrier densities (∆*p* > 10^20^ cm^−3^), thus a (de)increase in holes (observed at length scales greater than the Debye length) must correspond to an equal (in)decrease of ions to preserve charge neutrality^16^. Thus, the change in transmissivity corresponds to local ion motion^17,18^.

The light transmitted through the OMIEC is either directly captured to obtain a broadband transmission image (Fig. 1d) or passed through a slit aligned along the direction of ion motion to collect a one-dimensional (1D)-spatial, 1D-spectral image^19^. During (de)doping, the neutral chain absorption band in the visible range (1.7 to 2.4 eV) (in)decreases and the polaron absorption band (1.0 to 1.6 eV) (de)increases^11,20,21^. In the visible range, absorption is dominated by the neutral polythiophene backbone; thus, positive and negative differential transmissions correspond to doping and dedoping, respectively (Fig. 1d). Thus, the differential transmission spectra can be used to infer the structural properties of the CP chains^22–24^ being (de)doped during each time increment (Fig. 1e) and spatial position (Fig. 1f).

**Fig. 2:**
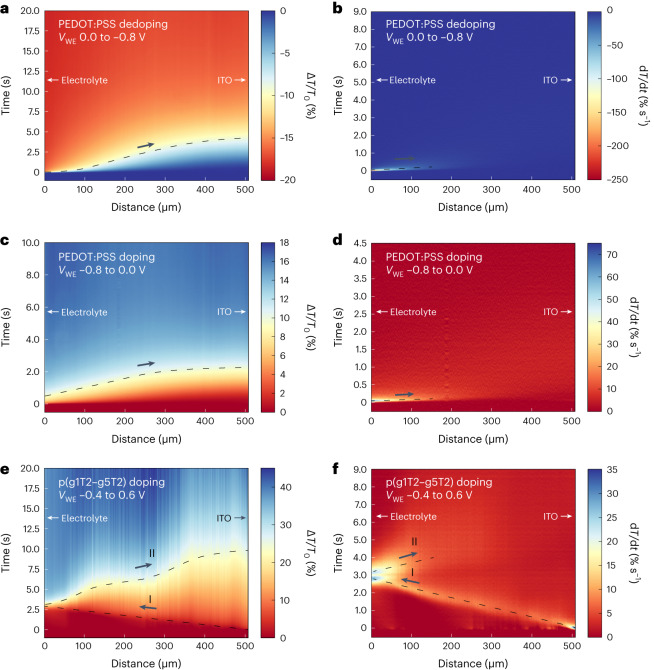
Electrochemical doping and dedoping in CPs. **a**,**b**, Spatiotemporal transmission (∆*T*/*T*_0_) profile (**a**) and corresponding time derivative (d*T*/d*t*) (**b**) during dedoping for PEDOT:PSS. **c**,**d**, Doping of PEDOT:PSS: spatiotemporal transmission (∆*T*/*T*_0_) profile (**c**) and corresponding time derivative (d*T*/d*t*) (**d**). **e**,**f**, Doping of p(g1T2-g5T2): spatiotemporal transmission (∆*T*/*T*_0_) profile (**e**) and corresponding time derivative (d*T*/d*t*) (**f**). Dashed lines are used to highlight the propagation of (de)doping fronts, and I and II denote the contact-to-electrolyte (type I) and electrolyte-to-contact (type II) doping fronts in p(g1T2-g5T2), respectively.

**Extended Data Fig. 1:**
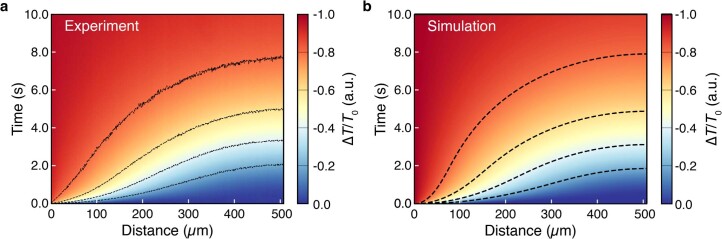
Simulated doping front from ionic drift-diffusion. Comparison of the **a**, experimental and **b**, simulated time dependent transmission ∆*T*/*T*_0_ for dedoping of PEDOT:PSS (*V*_WE,i_ = 0.0 V to *V*_WE,f_ = −0.8 V *vs*. Ag/AgCl) with contour lines showing transitions across different ∆*T*/*T*_0_ values (−0.2, −0.4, −0.6, and −0.8). The simulation shows excellent agreement with the experimental results. Simulation details are discussed in Supplementary Note 1.

The spatiotemporal transmission (∆*T*/*T*_0_) during dedoping of PEDOT:PSS (from an initial working electrode potential *V*_WE,i_ = 0 to a final working electrode potential *V*_WE,f_ = −0.8 V versus Ag–AgCl) is visualized as a 2D heatmap with distance along the film on the *x* axis and time after applying a potential on the *y* axis (Fig. 2a). The dedoping front is well described by a 1D transport model for ions, where holes can be ignored due to their higher mobility (Extended Data Fig. 1). Initially, the applied potential causes holes to drift towards the ITO contact, leading to a large electric field at the OMIEC–electrolyte interface. The field drives cations from the electrolyte into the OMIEC, leading to a rapid decrease in ∆*T*/*T*_0_ near the polymer–electrolyte interface. The time derivative of transmission (d*T*/d*t*) (Fig. 2b) highlights the region with the highest rate of local dedoping (where ion and hole currents converge). Further ionic drift screens and broadens the electric field, leading to a broader ∆*T*/*T*_0_ profile and diminished d*T*/d*t* near the ITO contact. The sigmoidal shape of ∆*T*/*T*_0_ emerges from the ions reaching and subsequently accumulating at the ITO contact. Similarly, when doping PEDOT:PSS from its dedoped state, an accumulation region forms near the polymer–electrolyte interface where cations are subsequently expelled out of the film rapidly, leading to a sharp increase in transmission near the electrolyte at early timescales (Fig. 2c,d).

We compare the ion dynamics of PEDOT:PSS to the state-of-the-art OMIEC p(g1T2-g5T2)^11^. In contrast to PEDOT:PSS, doping of p(g1T2-g5T2) (*V*_WE,i_ = −0.4 V to *V*_WE,f_ = 0.6 V versus Ag–AgCl) consists of negatively charged anions compensating holes, leading to increased transmission (Fig. 2e). Unlike PEDOT:PSS, doping of p(g1T2-g5T2) does not initiate at the OMIEC–electrolyte interface where ions can enter the film, and instead holes accumulate at the ITO contact where they are injected. This ‘reverse doping front’ propagates from the ITO contact towards the OMIEC–electrolyte interface (type I doping) and, after reaching the interface, propagates back towards the ITO contact (type II doping). This phenomenon is highlighted in the d*T*/d*t* heatmap (Fig. 2f), which shows localized doping at the ITO contact at early timescales. However, rather than broadening with time, the location of peak d*T*/d*t* moves from the ITO contact towards the electrolyte (I). Then, during the type II doping front, ∆*T*/*T*_0_ and d*T*/d*t* broaden with time and distance similar to doping of PEDOT:PSS (Fig. 2e,f).

## Origin of the type I doping front

**Fig. 3:**
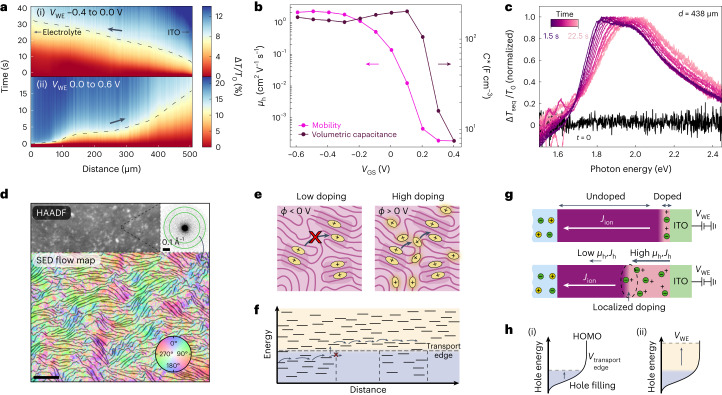
Origin of the type I doping front. **a**, Isolation of the type I and type II doping fronts by the changing initial and final doping level. **b**, Voltage-dependent hole mobility (*µ*_h_) and volumetric capacitance (C^*^). **c**, Sequential differential transmission (∆*T*_seq_/*T*_0_) spectra for ∆*t* = 1.5 s time increments during the moving front experiment (*V*_WE,i_ = −0.4 to *V*_WE,f_ = 0.6 V versus Ag–AgCl), where ∆*T*_seq_ = *T*(*t*) − *T*(*t* − ∆*t*). **d**, Scanning transmission electron microscopy of p(g1T2-g5T2) with high-angle annular darkfield (HAADF) image and SED polymer flow map where the colour corresponds to the spatial orientation of the CP chain alignment direction and the intensity corresponds to the local diffraction intensity. Scale bar, 100 nm. **e**, Schematic describing the origin of low mobility states where at low doping levels, holes are confined to ordered regions of the microstructure corresponding to **f**, low energy hole states, but as doping density increases, holes occupy higher energy states in less ordered regions and conduct more efficiently. **g**, The low *µ*_h_ and therefore low hole drift *J*_h_ through the poorly doped polymer results in this scenario, doping initiating at the contact, followed by propagation of the moderately doped region towards the electrolyte during the hole-limited doping front (type I). *J*_ion_ denotes the direction of net positive charge carried by ions (both Na^+^ expulsion and Cl^−^ intercalation). **h**, The hole-limited doping front (i) results in filling of hole sites up to the transport edge, after which the doped region propagates in space towards the electrolyte. Then, during the type II doping (ii), front hole sites are filled to the applied energy level (*V*_WE_).

By probing doping of p(g1T2-g5T2) with varied *V*_WE,i_ and *V*_WE,f_, we find that we can isolate the type I and type II doping processes (Fig. 3a). Doping fronts from reducing potentials (when the CP is poorly doped, *V*_WE,i_ = −0.4 V versus Ag–AgCl) to moderate doping levels (*V*_WE,f_ = 0 V versus Ag–AgCl) only propagate from the ITO contact towards the electrolyte (Fig. 3a,(i)), whereas further doping propagates from the electrolyte to the ITO contact (Fig. 3a,(ii)). This result indicates that the type I doping front only occurs when doping from low hole concentrations.

**Extended Data Fig. 2:**
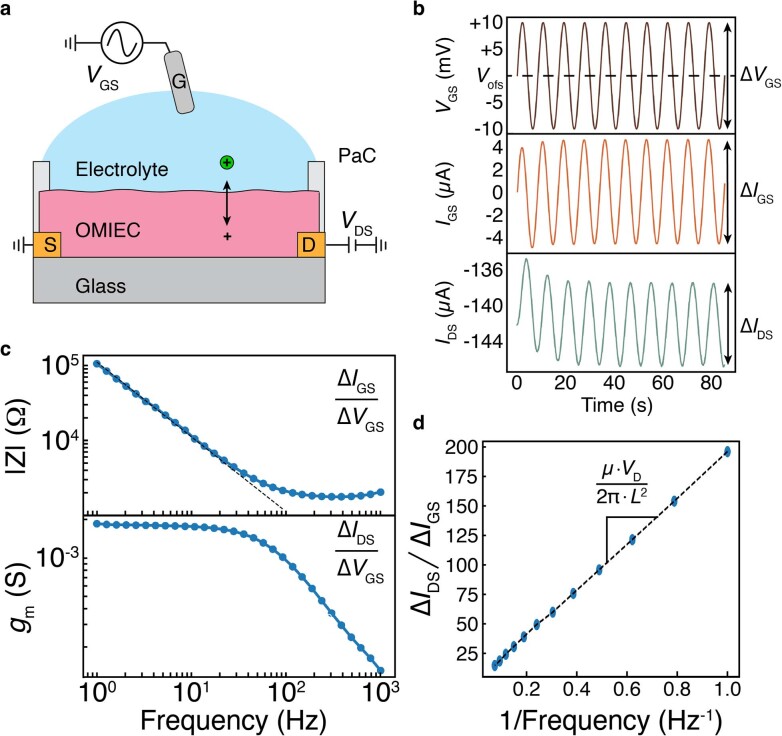
Voltage-dependent mobility measurements. Schematic experimental setup (gate electrode not drawn to scale) **a**, and example resulting data **b**, for the voltage dependent mobility measurement. An oscillating sine wave voltage with amplitude of ±10 mV is applied to the gate. The frequency dependent impedance and transconductance **c**, can be extracted from the gate current and drain current divided by the amplitude of the gate voltage sine wave. The mobility can be extracted by **d**, fitting a straight line to the ratio of drain current and gate current amplitudes as a function of the inverse frequency.

Often changes in hole concentration lead to sharp changes in hole mobility, *µ*_h_, in CPs^25^. To probe the effect of hole concentration on *µ*_h_, we use a voltage-dependent *µ*_h_ measurement using p(g1T2-g5T2) OECTs as described in Extended Data Fig. 2. Note that the polarity of the gate voltage (*V*_GS_) for OECTs is inverted compared to *V*_WE_ in the moving front devices since the potential is applied to the Ag–AgCl electrode rather than to the contact. We find a sharp drop in *µ*_h_ at a *V*_GS_ near 0 V, which matches the doping level corresponding to the transition from type I to type II doping (Fig. 3b). By contrast, the volumetric capacitance (C^*^), which is related to the density of available hole states at a particular Fermi level, drops at higher *V*_GS_ of approximately 0.3 V. The offset in the decay of *µ*_h_ and C^*^ indicates that there is a large concentration of low *µ*_h_ hole states at the edge of the highest occupied molecular orbital level that must be occupied before high *µ*_h_ hole states are occupied, resulting in an apparent transport edge^26^ near 0 V.

The differential transmission spectra of p(g1T2-g5T2) near the ITO contact during doping shows the spectral shape CP chains doped during subsequent time increments (Fig. 3c). As the CP is doped, the neutral chain absorption band is bleached giving a positive ∆*T*/*T*_0_ feature in the visible range, which corresponds to the absorption of the population of chains doped during each time increment. The hole sites doped during the initial timestep show relatively strong vibronic character indicative of high conjugation length thiophene backbones^23,27,28^. With increasing time, the spectra blueshift and the vibronic character weaken, indicating that with increasing doping, the CP chains doped exhibit a lower degree of conjugation along the backbone. Thus, the straightest or highest order polymer chains comprise the lowest energy, lowest *µ*_h_ hole sites.

We use scanning electron diffraction (SED) imaging of p(g1T2-g5T2) with a transmission electron microscope to observe diffraction from interchain ordering (π–π stacking), giving a spatially resolved picture of local CP chain alignment^29^. In general, the polymer is weakly aligned, but the CP chain alignment flow map reveals semicrystalline domains with preferred chain orientation ranging from 20 to 100 nm in size (Fig. 3d). These domains of relatively high CP ordering are isolated, separated in space by near-amorphous regions with poor chain alignment.

The changes in transmission ∆*T*/*T*_0_ during the type I doping front (approximately 10%) correspond to changes in local hole concentration of approximately 2.4 × 10^20^ cm^−3^ and therefore must correspond to anion–hole pairs to preserve local electroneutrality (Supplementary Note 2). Thus, local changes in transmission correspond to where ionic and electronic currents converge along the film length, analogous to microscopic mapping of electroluminescence in light-emitting electrochemical cells^30,31^.

On initial application of *V*_WE,f_ we expect a linear potential drop across the length of the film. The location where doping occurs then depends on the relative magnitudes of the ionic and electronic conductivities (*σ*_ion_ and *σ*_h_, respectively). Thus, the initiation of doping at the ITO contact indicates an initially higher *σ*_ion_ compared to *σ*_h_. In many glycolated semiconducting polymers, there are likely to be ion pairs present in the bulk of the material when it is in the dedoped state^32^ and doping can occur either by cation expulsion (similar to doping of PEDOT:PSS) or by anion intercalation^33^. Thus, the larger *σ*_ion_ compared to *σ*_h_ does not necessarily indicate a higher ion mobility, *µ*_ion_, compared to *µ*_h_. Instead, it probably results from the combination of the high ion concentration, *C*_ion_ (both cations and anions), as well as the low initial hole concentration and low *µ*_h_.

At the start of doping, the lowest energy hole sites that correspond to ordered CP chains are doped first. The spatial separation between these ordered regions results in low *µ*_h_ due to the lack of a percolating network of accessible hole sites at similar energies (Fig. 3e). As the doping level increases, the higher energy hole sites in disordered regions begin to fill, resulting in a more spatially and energetically uniform hole landscape (Fig. 3f) and therefore higher *µ*_h_.

**Extended Data Fig. 3:**
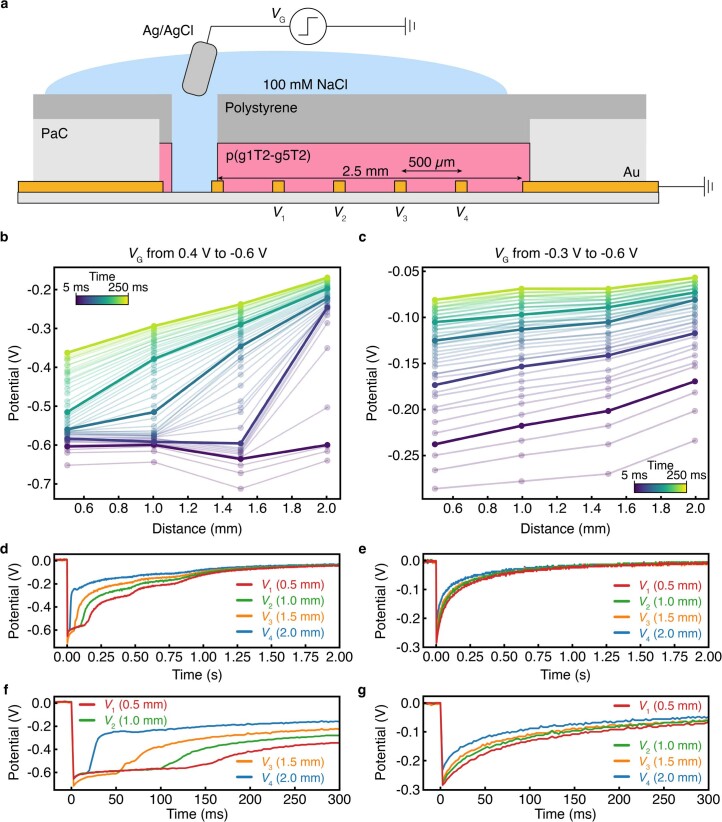
Internal channel potential profile evolution during doping front experiments. **a**, Schematic of device structure for internal potential measurements at inserted probe electrodes (V_1-4_) at increasing distance from the electrolyte/polymer interface with 500 µm spacing. Internal voltage profile measurements for devices operated in the **b**, Type I doping front regime (*V*_G_ from 0.4 V to −0.6 V) and **c**, the Type II doping front regime (*V*_G_ from −0.3 V to −0.6 V). We note that potentials are reversed compared to optical experiments as they are applied to the Ag/AgCl. The potential for each probe, measured with respect to the grounded gold contact at the end of the channel, is plotted as a function of time for both the Type I **d**, and Type II **e**, regimes, where the beginning of the transients show an initial delay before the potential rises when doping occurs in the **f**, Type I doping regime, whereas there is no delay in the potential rising for the **g**, Type II doping regime.

At the device scale, the region near the ITO contact is doped first due to the higher *σ*_ion_ compared to *σ*_h_ (Fig. 3g). Drift of ions leaves a negatively charged space charge region near the hole injecting contact. The injected holes drift slowly due to the low *µ*_h_ and remain fixed near the contact until holes fill to the transport edge. Once the transport edge is reached, holes can move between the contact and region where ion and hole drift currents converge (where d*T*/d*t* peaks), causing the location of doping to shift away from the ITO contact. The position of the localized doping region moves linearly with time towards the OMIEC–electrolyte interface, where the rate of propagation of the doping front depends on the time to locally fill hole sites to the transport edge (Fig. 3h). Finally, once the type I doping front reaches the electrolyte–polymer interface, the more rapid type II doping proceeds until the CP is doped to the *V*_WE_ level. Measurements of the internal electric fields across the OMIEC film are included in Supplementary Note 3 and Extended Data Fig. 3.

**Extended Data Fig. 4:**
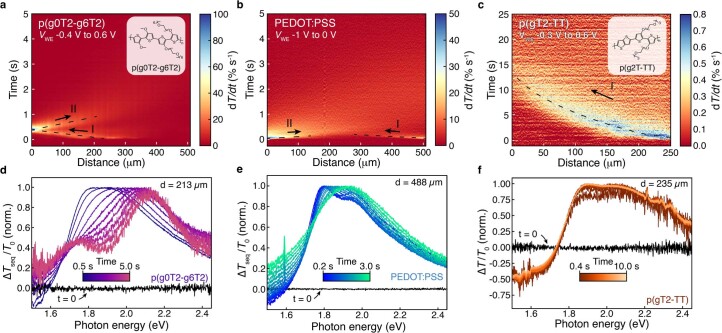
Hole-limited doping in other conjugated polymers. Transmission time derivative (d*T*/d*t*) for electrochemical doping of **a**, p(g0T2-g6T2) (*V*_WE,i_ = −0.4 V to *V*_WE,f_ = 0.6 V *vs*. Ag/AgCl), **b**, PEDOT:PSS (*V*_WE,i_ = −1.0 V to *V*_WE,f_ = 0.0 V *vs*. Ag/AgCl). and **c**, p(g2T-TT) (*V*_WE,i_ = −0.3 V to *V*_WE,f_ = 0.6 V *vs*. Ag/AgCl), and **c**, Sequential differential transmission spectra (∆*T*_seq_/*T*_0_ where ∆*T*_seq_ = *T*(*t*) − *T*(*t*-∆*t*)) for doping of **d**, p(g0T2-g6T2) (∆*t* = 500 ms, *d* = 213 µm) and **e**, PEDOT:PSS (∆*t* = 200 ms, *d* = 488 µm). Differential transmission spectra for **f**, p(g2T-TT) (∆*t* = 400 ms, *d* = 235 µm).

**Extended Data Fig. 5:**
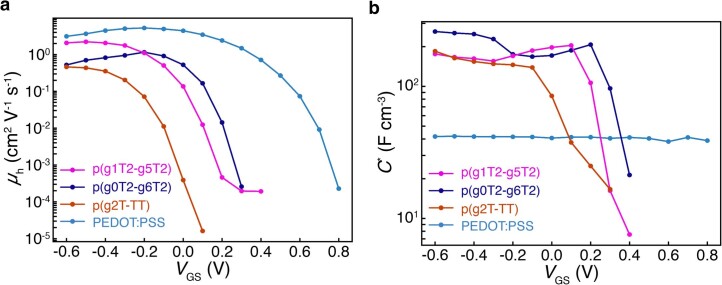
Mobility and volumetric capacitance vs. voltage. Comparison of voltage dependent mobilities **a**, and volumetric capacitances **b**, for p(g1T2-g5T2), p(g0T2-g6T2), p(g2T-TT), and PEDOT:PSS using OECTs (*W* = 800 µm, *L* = 100 µm). An Ag/AgCl electrode was used for the gate as described in the **Methods** section.

**Extended Data Fig. 6:**
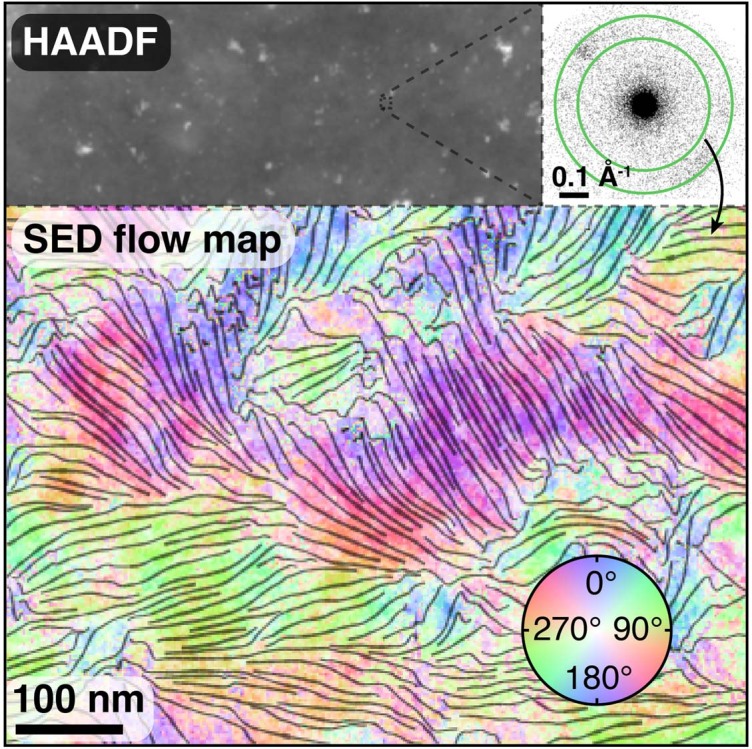
Scanning electron diffraction image of p(g0T2-g6T2). Transmission electron microscopy of p(g0T2-g6T2) with high-angle annular darkfield (HAADF) image and scanning electron diffraction (SED) polymer flow map where the colour corresponds to the spatial orientation of the CP chain alignment direction and the intensity corresponds to the local diffraction intensity.

Heterogeneous disorder is common among CPs^34^ and therefore we expect that hole-limited doping is not specific to p(g1T2-g5T2). To test the generality of this phenomenon, we measured the doping fronts for p(g0T2-g6T2)^11^, p(g2T-TT) and PEDOT:PSS (Extended Data Fig. 4). All polymers tested show evidence of a hole-limited doping regime at low hole concentrations (Supplementary Note 4) and show a drop in *µ*_h_ as the carrier density is reduced (Extended Data Fig. 5). The timescale of hole-limited doping is shorter for p(g0T2-g6T2) compared to p(g1T2-g5T2). SED imaging of p(g0T2-g6T2) shows larger regions (up to 1 μm) of aligned CP chains with stronger π–π ordering than p(g1T2-g5T2). As a result, there is a reduced volume fraction of amorphous regions and greater connectivity of ordered domains (Extended Data Fig. 6), reducing the effect of hole-limited doping. Conversely, PEDOT:PSS has been shown to have a relatively low activation energy for transport and efficient tunnelling over distances greater than 2 nm (ref. ^35^), indicating a homogenous energy landscape for holes despite microstructural heterogeneity.

## Operando electrochemical transistor characterization

**Fig. 4:**
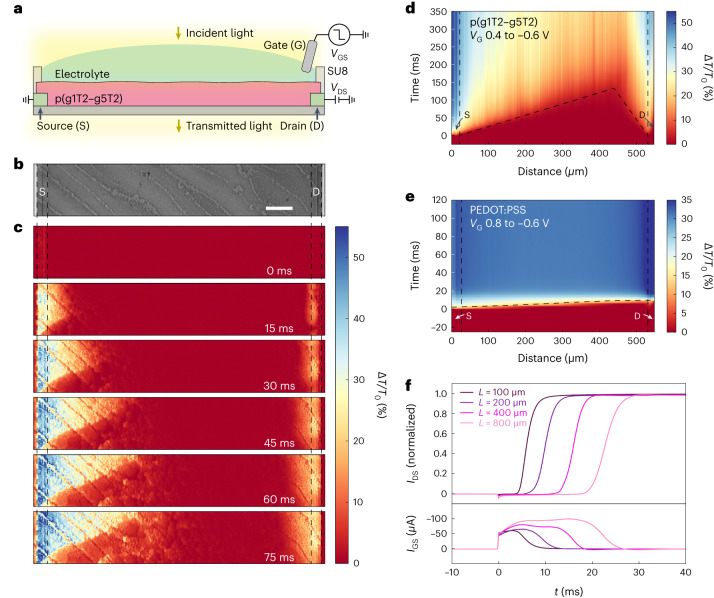
Operando transmission microscopy of OECTs. **a**, Schematic of the experimental setup (the Ag–AgCl electrode is not drawn to scale). **b**, Optical transmission image of the device (greyscale). Scale bar, 50 μm. **c**, Time-resolved differential transmission images of electrochemical doping from the intrinsic (*V*_GS_ = 0.4 V) to the fully doped (*V*_GS_ = −0.6 V) state. **d**,**e**, Spatiotemporal map of transmission intensity for doping of p(g1T2-g5T2) (**d**) and PEDOT:PSS (**e**) electrochemical transistors with dashed lines highlighting the doping fronts (from *V*_GS_ = 0.8 V to *V*_GS_ = −0.6 V, *V*_DS_ = −0.6 V). **f**, Drain (upper) and gate (lower) current (*I*_DS_ and *I*_GS_, respectively) transients during electrochemical doping of a pg1T2-g5T2 OECT with increasing channel length (*W* = 100 µm). Vertical dashed lines in **b**–**e** indicate the source (S) and drain (D) contacts.

**Extended Data Fig. 7:**
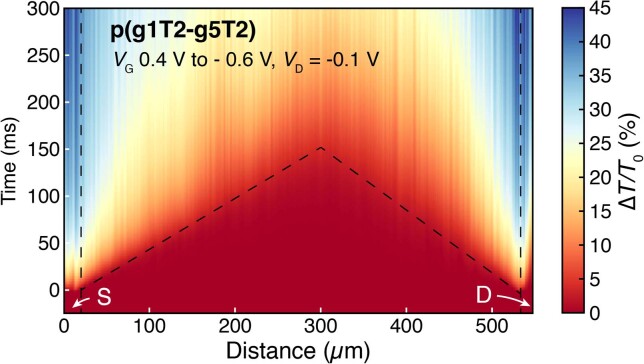
Doping of a p(g1T2-g5T2) OECT at lower drain bias. At a lower drain-source bias (*V*_DS_ = −0.1 V), the meeting point of the doping fronts in *operando* measurements of OECTs moves closer to the centre of the channel compared to the OECT operated at *V*_DS_ = −0.6 V (Fig. 4d in the Main Text). Vertical dashed lines indicate the source (S) and drain (D) contacts.

**Extended Data Fig. 8:**
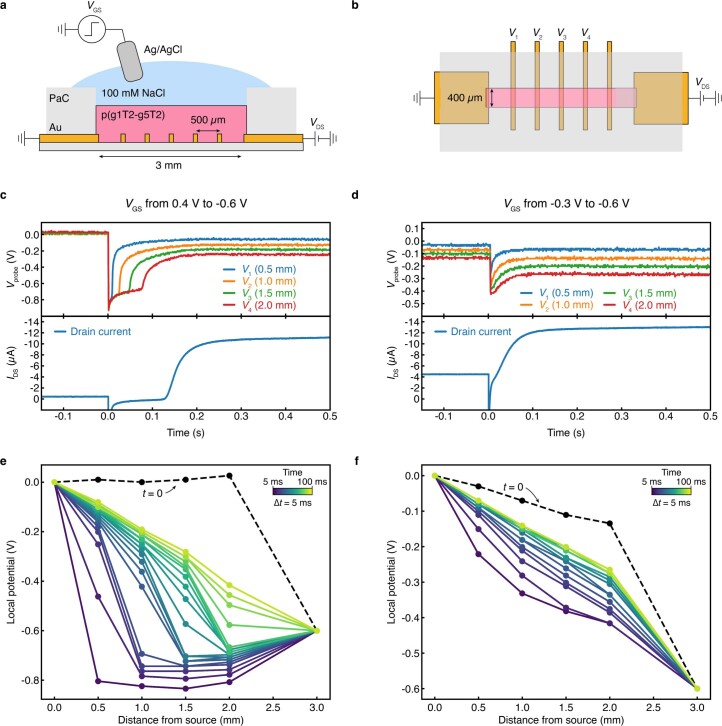
Internal channel potential profile evolution during switching of an OECT. We measured the internal potential (with respect to the grounded source electrode) along the length of a p(g1T2-g5T2) channel (*L* = 3 mm, *W* = 0.4 mm) using inserted voltage probes (30 µm width, 0.5 mm spacing) along the channel as schematically depicted in **a** and **b** (note that the gate is not drawn to scale in **a**). The sample was using the same methodology described for OECT fabrication described in the methods section. Potentials were measured at each probe (*V*_1-4_) using a 4-channel oscilloscope (Keysight DSOX1204G) while a source-measure unit (Keysight B2902A) was used to apply a pulse between the Ag/AgCl gate and the gold contact. We note that the voltage probes measure the electrochemical potential of electronic charges, which includes contributions due to the local electric potential as well as shifts in the Fermi level resulting from doping. The resulting transient potential profiles **c**, in the hole-limited doping regime (*V*_G_ from 0.4 V to −0.6 V) show a delay in the rise in potential for voltage probes further from the source electrode, closely corresponding with the turn on delay time for the OECT source-drain current (*I*_SD_). In contrast, when doped in the standard regime **d**, (*V*_G_ from −0.3 V to −0.6 V) the potentials at each probe rise simultaneously and *I*_SD_ increases immediately, indicating efficient transport of holes along the channel. The **e**, potential profiles along the channel during hole-limited doping additionally show that the region of largest potential drop moves from the source electrode towards the drain, comparable to the movement of the doping front observed in Fig. 4d. In contrast, when the film is initially partially doped **f**, the potential remains nearly linear along the channel throughout the measurement.

The impact of hole-limited doping in p(g1T2-g5T2) OECTs is measured using operando transmission microscopy (Fig. 4a,b) during switching from the off state (*V*_G_ = 0.4 V) to the on state (*V*_G_ = −0.6 V). On gating, the regions nearest the contacts are doped first, and doping proceeds from the contacts towards the centre of the channel with increasing time (Fig. 4c,d), corresponding to type I (hole-limited) doping. The doping front originating from the source travels much faster than from the drain due to the applied drain-source voltage (*V*_DS_ = −0.6 V). When *V*_DS_ is lowered to −0.1 V, the meeting point of the source and drain doping fronts shifts closer to the centre of the channel (Extended Data Fig. 7). Voltage measurements along the channel show that the largest potential drop during hole-limited doping is across the boundary between the moderately and poorly doped regions (Extended Data Fig. 8). In contrast to p(g1T2-g5T2), there is very little spatial variation in the doping rate of PEDOT:PSS OECTs during switching (Fig. 4e). The lack of spatial dependence during doping indicates that mobile holes rearrange under the applied field much faster than ions, leading to spatially uniform (type II) doping.

**Extended Data Fig. 9:**
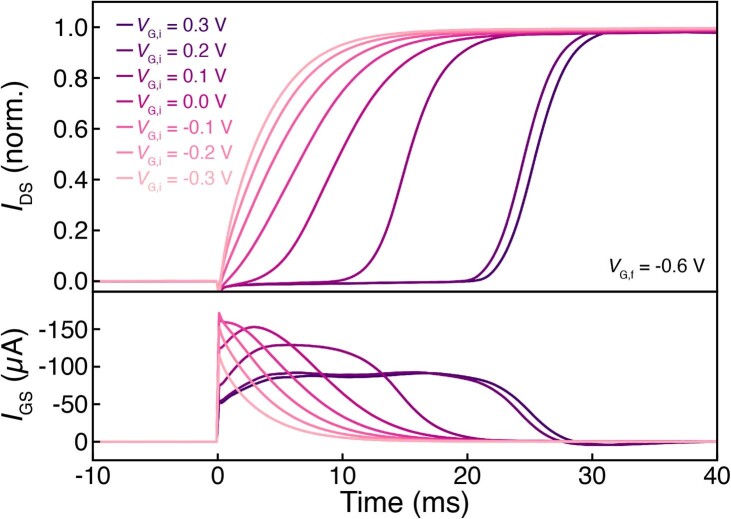
OECT rise times for varied initial gate voltages. The drain current rise times were measured from transitions from varied initial gate voltage (*V*_G,i_) to the fully doped state (*V*_G,f_ = −0.6 V) for p(g1T2-g5T2) OECTs (*L* = 800 µm, *W* = 100 µm, *V*_DS_ = −0.6 V). Drain-source currents are normalized between 0.0 and 1.0 for ease of comparison.

**Extended Data Fig. 10:**
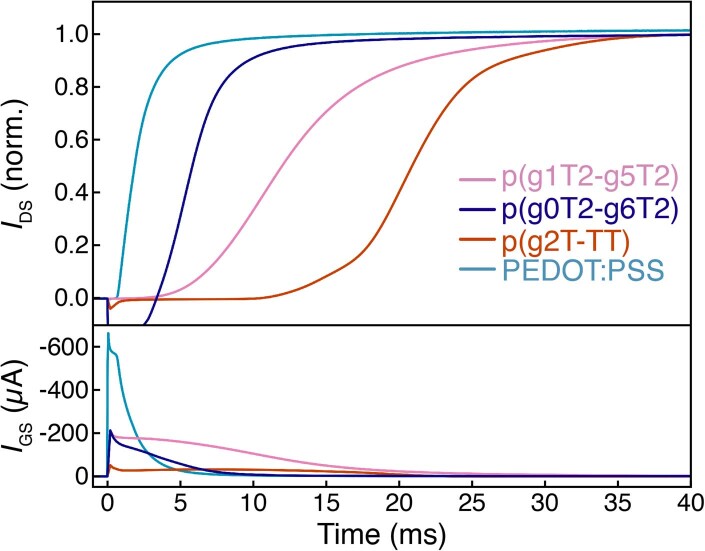
Switching time delay for OECTs using different OMIEC channel materials. Comparison of the switching time delays for p(g1T2-g5T2) (pink), p(g0T2-g6T2) (purple), p(g2T-TT) (orange), and PEDOT:PSS (blue) OECTs (*W* = 800 µm, *L* = 100 µm, *V*_DS_ = −0.6 V) showing hole-limited doping delay of approximately 8 ms, 4 ms, 15 ms to 20 ms, and 0.5 ms, respectively. Both p(g1T2-g5T2) and p(g0T2-g6T2) OECTs were switched from *V*_GS,i_ = 0.4 V to *V*_GS,f_ = −0.6 V, the p(g2T-TT) OECT was switched from *V*_GS,i_ = 0.3 V to *V*_GS,f_ = −0.6 V, and the PEDOT:PSS OECT was switched from *V*_GS,i_ = 0.8 V to *V*_GS,f_ = −0.6 V.

Hole-limited doping leads to a delay during switching of the OECT (Fig. 4f), which is about two orders of magnitude larger than the switching time predicted using the typical ion equivalent circuit model^36^. Because the doping front must traverse the channel, this switching time delay is lowered by reducing the channel length. The behaviour also shows a strong dependence on the initial potential (*V*_i_), where the switching time delay is only observed for OECTs switched from below the transport edge (*V*_GS_ = 0 V), and the delay time depends on the initial potential (Extended Data Fig. 9). This switching delay from hole-limited doping is critical for OMIEC-based devices that require operating frequencies ranging from 10 kHz to 1 MHz such as neural recording electrodes^2^ and neuromorphic devices^37^. In contrast to p(g1T2-g5T2), for OECTs using p(g0T2-g6T2) and PEDOT:PSS channel materials that have less heterogeneous disorder, the switching delay times due to hole-limited doping are shorter (Extended Data Fig. 10).

## Discussion

Using a newly developed microscopic characterization methodology, we have demonstrated a hole-limited doping mechanism for charging of OMIECs. This hole-limited doping is the result of heterogeneous disorder of mixed conducting polymers that results in a threshold doping concentration for efficient conduction. Notably, the poor transport at low doping levels does not harm *µ*_h_ in fully doped OMIEC films, indicating that benchmarking the performance of OMIECs in their doped state is insufficient. By measuring devices during realistic operating conditions and at relevant timescales, we find that the hole-limited doping has a substantial effect on the speed of OECT operation in the subthreshold regime.

The results show that design principles for traditional organic electronics do not necessarily translate to mixed conducting devices. While order is usually expected to improve electronic transport, in electrochemical devices that require lower order regions to facilitate ion transport, heterogeneous order can have a detrimental effect on performance. This difference probably results from the lower electric fields used in OECTs, limiting the ability of holes to hop over barriers^38^, as well as holes sampling sites throughout the volume of the microstructure rather than being confined to a 2D interface as is the case in field-effect devices. Instead, homogeneous order is optimal for mitigating hole-filling delays in device switching without disrupting ion transport. Controlling microstructural heterogeneity provides a pathway for overcoming the previously overlooked fundamental limit for device speed caused by hole-limited doping. We expect the presented framework will aide in the interpretation of classical electrical and electrochemical measurements to differentiate between ionic and electronic effects more accurately in operating mixed conductor devices.

## Methods

### Preparation of conducting polymer solutions

The PEDOT:PSS dispersion was prepared by mixing Clevios PH1000 (Heraeus) with 6% v/v ethylene glycol and 1% v/v (3-glycidyloxypropyl)trimethoxysilane, sonicating for 10 min and then filtering through a 0.45 µm polyvinylidene fluoride syringe filter. The p(g1T2-g5T2) solution was prepared by dissolving p(g1T2-g5T2) in chloroform (5 mg ml^−1^) and stirring at room temperature overnight.

### Device fabrication

Fabrication of transparent samples for optical microscopy started with 10 min of sonication of ITO coated boro-aluminosilicate glass wafers (University Wafer 2544) submerged in acetone followed by isopropyl alcohol (IPA). The contact layer was patterned by coating wafers with AZ 4533 positive photoresist (Microchemicals) (spin coated at 4,000 rpm for 45 s, acceleration of 500 rpm s^−1^, soft baking at 100 °C for 50 s) followed by ultraviolet (UV) exposure (198 mJ cm^−2^, Karl Suss MA6 Mask Aligner) and development in AZ 726 MIF developer (Microchemicals) for 100 s. The pattern was baked at 115 °C for 50 s to improve adhesion between the resist and substrate. ITO was etched using a dilute aqua regia ITO etchant solution (A-Gas) for 1 h and the remaining resist was stripped by washing the substrate with acetone. Conducting polymers were coated onto the patterned ITO wafers (spin coating at 1,000 rpm for 40 s, acceleration of 500 rpm s^−1^). The resulting film thicknesses were 224 ± 10, 111 ± 12, 66 ± 12 and 52 ± 6 nm for PEDOT:PSS, p(g1T2-g5T2), p(g0T2-g6T2) and p(g2T-TT), respectively. PEDOT:PSS coated wafers were baked at 120 °C for 20 min for crosslinking of GOPS and soaked in deionized water overnight to remove excess PSS molecules. The conducting polymer was patterned into 200 µm-wide, 522.5 µm-long channels (10 µm overlap with ITO contact) using photolithography by coating with AZ 5214E image reversal resist (Microchemicals) (spin coating at 2,500 rpm for 45 s, acceleration of 500 rpm s^−1^, soft baking at 115 °C for 120 s) followed by UV exposure (180 mJ cm^−2^) and development in AZ 726 MIF developer for 20 s. The polymer was defined by reactive ion etching (recipe, Plasma Pro 80 RIE, Oxford Instruments) and the remaining resist was stripped by washing with acetone. The films were exposed to 0.1 M NaCl to allow for passive swelling and uptake of ions. The SU8 capping layer was defined with photolithography by coating the wafers with SU8 2000.5 (Kayaku Advanced Materials, Inc.) (500 rpm for 10 s, acceleration of 100 rpm s^−1^ followed by 2,000 rpm for 30 s, acceleration of 300 rpm s^−1^, then soft baking at 95 °C for 60 s) followed by UV exposure (60 mJ cm^−2^) and postexposure bake at 95 °C for 90 s. The pattern was developed in propylene glycol monomethyl ether acetate (PGMEA) for 75 s then rinsed with PGMEA then IPA followed by a hard bake at 120 °C for 20 min to anneal the SU8 layer. Finished wafers were diced with a diamond scribe and tile cutter tool and a silicone well was defined using an adhesive-backed silicone (McMaster-Carr) to confine the electrolyte.

Fabrication of OECTs for device characterization started with 10 min of sonication of borosilicate glass wafers (900 µm-thick, double-side polished Microchemicals) submerged in acetone followed by IPA then baked at 150 °C to remove any residual moisture. Gold contacts were patterned using metal lift off, which consisted of coating with AZ nLoF 2035 negative photoresist (Microchemicals) (spin coating at 500 rpm for 5 s, acceleration of 1,000 rpm s^−1^, followed by 3,000 rpm for 45 s, acceleration of 8,000 rpm s^−1^, then soft baked at 110 °C for 60 s) followed by UV exposure (60 mJ cm^−2^), a postexposure bake (110 °C for 180 s) and development in AZ 826 MIF (Microchemicals) for 30 s. The patterned wafer was coated with 5 nm of titanium then 100 nm of gold (E-beam Evaporator, Kurt J. Lesker Company), and lift off was performed by submerging the wafer in NI555 stripper (Microchemicals) for 10 h followed by rinsing with acetone then IPA. The patterned metal coated wafers are then coated with a parylene bilayer by first treating the wafer with oxygen plasma for 60 s followed by submerging the wafer in a dilute silane solution (3% v/v A174 silane dissolved in 0.1% v/v acetic acid in deionized water) for 45 s to improve parylene adhesion to the wafer. The silane treated wafer is rinsed with ethanol and heated for 1 h at 75 °C then coated with a 2 µm layer of parylene (PDS 2010 Labcoter 2, Specialty Coating Systems) followed by coating with a soap surfactant layer (2% v/v Micro 90 soap in deionized water spin coated at 1,000 rpm for 30 s and dried in air for 20 min) followed by a second deposition of a 2 µm layer of parylene. The trenches for depositing polymer channels were defined in the parylene bilayer with photolithography by coating with AZ10XT positive resist (Microchemicals) (spin coating at 3,000 rpm for 45 s, acceleration of 8,000 rpm s^−1^, soft baking at 115 °C for 120 s) followed by UV exposure (540 mJ cm^−2^) and developing in AZ 726 MIF developer (Microchemicals) for 10 min. Then, trenches were etched using reactive ion etching (recipe) and the wafers were diced with a diamond scribe and tile cutter tool, and conducting polymers were coated using the same spin coating parameters described above. The conducting polymer channel was defined by peeling off the top parylene layer using Kapton tape, leaving conducting polymer only in the patterned trench. The OECTs were annealed at 120 °C for 20 min to match the annealing procedure for moving front devices. A silicone well was defined using an adhesive-backed silicone (McMaster-Carr) to confine the electrolyte.

### Optical transmission microscopy

Optical microscopy was performed with a home-built inverted widefield transmission microscope. The sample was illuminated from the top using a broadband light source (Fiber-Lite DC-950) focused onto the sample with an OSL2 fibre bundle focusing package (ThorLabs OSL2FOC). The sample was fixed to a piezo-driven sample stage (Attocube ECSx5050/AL/RT/NUM) to control the position of the sample in the *x*, *y* and *z* axes and the transmitted light was captured with a ×10 objective (Olympus PLN ×10 objective, 0.25 numerical aperture (NA)) that was fixed to the optical table. For greyscale imaging, light was imaged by a tube lens (300 mm fluorescence, visible-near-infrared coated achromat, Edmund Optics) onto the camera (FLIR, Grasshopper3, GS3-U3-23S6M-C). The magnification was ×16.7 (350 nm per pixel). The image acquisition was controlled using custom LabView code.

For spectral resolution, the imaging system was extended by a 1:1 telecentric lens pair (300 mm of fluorescence, visible-near-infrared coated achromat, Edmund Optics) to gain access to an intermediate image and back focal plane^19^. The transmitted light first passed through a diffraction-limited slit placed in the intermediate imaging plane. The slit was aligned along the long axis of the sample. Afterwards, an F2 prism (PS852, Thorlabs) placed in the intermediate back focal plane dispersed the transmitted light orthogonally to the slit before being imaged onto a 16-bit scientific CMOS camera (Hamamatsu, ORCA Flash 4 V3). The overall magnification was ×16.7 (390 nm per pixel) and spectral calibration was achieved through a series of bandpass filters (FBXXX-10, Thorlabs) across the visible spectral region. The image acquisition was controlled using custom LabView code.

### Optical data analysis

To analyse transmission image data, a background image (*T*_0_) was obtained by averaging 50 frames acquired before the application of the voltage pulse *V*_WE,f_. The subsequent frames were converted to differential transmission by first subtracting the background image (∆*T* = *T*[t] − *T*_0_) followed by dividing the difference by the background image *T*_0_, yielding ∆*T*/*T*_0_ images. To obtain spatiotemporal heatmaps, images were rotated to align the length of the sample with the *x* axis and cropped to omit the edges of the polymer film that can produce scattering artefacts. The intensity was averaged along the *y* axis to produce heatmaps. Derivative maps were obtained by numerical differentiation along the time axis for each distance increment.

Raw spectral images were calibrated using a series of bandpass filters to determine the associated location on the camera for each energy band. Then, a calibration line converting pixel number to energy was fit for each horizontal row of pixels on the basis of the linear relationship between photon energy and dispersion by the prism. Using the row-by-row calibration, the pixels of each row for the raw images were adjusted to a uniform energy scale along the *x* axis of the image. The energy-adjusted images were converted to differential transmission images by averaging 50 frames before the voltage pulse to constitute the background (*T*_0_) and then subtracting then dividing by *T*_0_ for all subsequent frames. Sequential spectra were obtained by averaging the ∆*T*/*T*_0_ spectra over 1.5 s intervals and subtracting ∆*T*/*T*_0_ from the previous interval, giving the change in transmission over the 1.5 s period of interest, *T*(*t*) − *T*(*t* − 1.5 s). For comparison of line shapes, the spectra were scaled by dividing by the maximum intensity value. For reference, unnormalized spectra for Fig. 3c are included in Supplementary Fig. 4.

### Electrochemistry

Chronoamperometry was performed using a Gamry Instruments Interface 1010E Potentiostat. The sample was connected to the working electrode and a cylindrical Ag–AgCl pellet electrode (2 mm diameter, 4 mm length) was used as the combined reference and counter electrode. The initial voltage *V*_WE,i_ was held for 5 min before applying the final voltage *V*_WE,f_, at which the moving front measurement was performed. All experiments were performed using 0.1 M NaCl dissolved in water as the electrolyte.

### Electrical characterization

Electrochemical transistors were characterized using a Keysight B2902A Source-Measure Unit controlled custom Python code. Mobility and capacitance measurements are described in detail in Extended Data Fig. 2. OECT rise times were measured by applying a square pulse to the cylindrical Ag–AgCl pellet (2 mm diameter, 4 mm length) gate electrode and while monitoring the drain-source current under a constant drain bias of −0.6 V. All experiments were performed using 0.1 M NaCl dissolved in water as the electrolyte.

### Film thickness measurements

Film thicknesses were measured using a DekTak XT Profilometer with a scan rate of 17 µm s^−1^ and a stylus force of 1 mg. Each sample was measured in six locations and averaged.

### Transmission electron microscopy

SED data on freestanding p(g1T2-g5T2) and p(g0T2-g6T2) films (95 ± 8.5 and 37 ± 4.0 nm-thick, respectively) were acquired on a Thermo Fisher Spectra 300 operated at 300 kV with a beam diameter of approximately 3 nm and a convergence semi-angle of approximately 0.8 mrad. The beam current was 20 pA and the frame rate was 1 ms per pattern, with a step size of 5.6 nm, leading to an electron dose of approximately 40 e/Å^2^. The expected damage threshold for p(g1T2-g5T2) is not precisely known, but the critical dose has been measured for similar semiconducting polymer P3HT and found to be approximately 18 e/Å^−1^. Data were acquired at doses above this value as previous data taken on polymers indicated that the critical dose in scanning transmission electron microscopy (STEM) may be considerably higher, and more data were acquired at lower dose, with no discernible change in diffraction patterns. The diffraction patterns were acquired using a single-chip Medipix3 direct electron detector with 256 by 256 pixels, a camera length of 580 mm and a $\varDelta s$ of 0.0028 Å^−1^, leading to a maximum scattering vector of 0.5 Å^−1^. Additional data were acquired at a lower camera length of 185 mm and a $\varDelta s$ of 0.0087 Å^−1^, leading to a maximum scattering vector of 1.1 Å^−1^. The bright spots in the high-angle annular darkfield image were due to Pd inclusions from a catalyst, the fraction of which was estimated to be approximately 250 parts per million.

## Online content

Any methods, additional references, Nature Portfolio reporting summaries, source data, extended data, supplementary information, acknowledgements, peer review information; details of author contributions and competing interests; and statements of data and code availability are available at 10.1038/s41563-023-01601-5.

## Supplementary information

Supplementary Information Supplementary Notes 1–4, Table 1 and Figs. 1–7.

## Data Availability

The data underlying the figures in the main text are publicly available from the University of Cambridge repository at 10.17863/CAM.96816. The datasets generated and/or analysed during the current study are available from the corresponding author upon request.

## Code availability

Code is available upon request from the corresponding authors.
