# Supplementary material for: Hole-limited electrochemical doping in conjugated polymers
Source: Nat Mater. 2023 Jul 6;22(9):1121–7. doi: 10.1038/s41563-023-01601-5 (PMC10465356; doi:10.1038/s41563-023-01601-5)
Supplement: Supplementary file 1 — Supplementary Notes 1–4, Table 1 and Figs. 1–7. [file 41563_2023_1601_MOESM1_ESM.pdf]

nature materials

Article

https://doi.org/10.1038/s41563-023-01601-5

Hole-limited electrochemical doping in conjugated polymers

In the format provided by the
authors and unedited

Supplementary Information for

Hole-limited electrochemical doping in conjugated polymers

Contents:

**Supplementary Note 1**	Details of ionic drift-diffusion simulation
**Supplementary Note 2**	Estimation of carrier density change during hole-limited doping.
**Supplementary Note 3**	Internal electric fields during Type I and Type II doping.
**Supplementary Note 4**	Generality of hole-limited doping in conjugated polymers
**Supplementary Table 1**	Ionic drift-diffusion simulation parameters
**Supplementary Figure 1**	Transmitted lamp spectrum.
**Supplementary Figure 2**	Electrochemical doping of p(g0T2-g6T2) from -0.6 V *vs.* Ag/AgCl.
**Supplementary Figure 3**	Hole-limited doping with varied cationic species.
**Supplementary Figure 4**	Hole-limited doping with varied anionic species.
**Supplementary Figure 5**	Sequential differential transmission spectra during doping of p(g1T2-g5T2) before normalisation.
**Supplementary Figure 6**	Length dependence for switching time transients in p(g2T-TT) OECTs.
**Supplementary Figure 7**	Relationship between the applied potential and transmitted intensity.
**References**	

Supplementary Note 1: Details of ionic drift-diffusion simulation

Simulations were performed using a finite difference method. The potential was initialised as a linear drop at the polymer/electrolyte interface to simulate the depletion region. The motion of ions over each time increment was calculated using the following 1D drift-diffusion equation for cation flux.

$$J_{Cat}(x) = -\left(\mu_{Cat}[Cat](x)\frac{dV(x)}{dx} + D_{cat}\frac{d[Cat](x)}{dx}\right) \qquad \textbf{(Equation S1)}$$

Where J_{cat} is the cation flux at each position x, μ_{cat} is the cation mobility in PEDOT:PSS, [*Cat*] is the cation concentration at each position x, dV/dx is the electric field at each position x, and D_{cat} is the diffusion coefficient of cations in PEDOT:PSS which was approximated using the Einstein relation (D_{cat} = 0.0259 V x μ_{cat}). The change in [*Cat*] at each space increment (x) along the length (L) was calculated using **Equation S2** as follows.

$$\Delta[Cat](x)(t + t_{step}) = t_{step}(J_{cat}(x - dx) - J_{cat}(x))/dx \qquad \textbf{(Equation S2)}$$

Where t_{step} is the length of the time increments and dx is the length of the space increments. The change in concentration of holes (Δp) was calculated by conserving local charge neutrality according to **Equation S3**.

$$\frac{\Delta p(x)}{t_{step}} = -\frac{\Delta[Cat](x)}{t_{step}} \qquad \textbf{(Equation S3)}$$

$V(x)$ was recalculated using the volumetric capacitance C^* of PEDOT:PSS which was held constant for the simulation (C^* = 40 F cm^{-3}) using **Equation S4**.

$$V(x) = V_{WE} - \frac{q(p_0 - p(x))}{C^*} \qquad \textbf{(Equation S4)}$$

The relative change in transmission ($\Delta T/T_0$) of PEDOT:PSS is on the order of 20%, thus the linear approximation (Beer's Law) can be made (the visible light absorption is linearly proportional to the concentration of neutral PEDOT chains). Thus, the relative change in transmission ($\Delta T/T_0$) was estimated using the concentration of absorbing neutral chains, which is inversely proportional to p as described in **Equation S5**.

$$-\frac{\Delta T}{T_0} \propto \frac{p_0 - p(x)}{p_0} \qquad \textbf{(Equation S5)}$$

Finally, the transmission was rescaled to set the minimum $\Delta T/T_0$ to 0 and maximum $\Delta T/T_0$ to 1 for comparison with the experimental results.

A summary of the physical constants used in the simulation is summarised in **Table S1**.

Supplementary Note 2: Estimation of carrier density change during hole-limited doping.

During the Type I doping front, the transmission intensity $\Delta T/T_0$ of the doped region reaches approximately 10%, and $\Delta T/T_0$ in the fully doped state is approximately 44 %. To estimate the final doping level, we can integrate the voltage-dependent volumetric capacitance from the initial voltage ($V_{WE,i}$ = -0.4 V *vs.* Ag/AgCl) to the final voltage ($V_{WE,f}$ = 0.6 V *vs.* Ag/AgCl) giving a differential charge density of 146 C cm^{-3}, or hole concentration of approximately 9.1×10^{20} cm^{-3}. If we use a single absorption cross section to model the broadband transmission in this system, we expect the differential transmission to scale according to the following **Equation S6**.

$$\frac{\Delta T}{T_0} + 1 = e^{\Delta p \alpha L} \qquad \textbf{(Equation S6)}$$

Where Δp is the differential charge carrier density and α is the absorption cross section of the neutral CP chains, and L is the path length through the film which remains fixed. Thus, we can estimate that the Type I doping front corresponds to doping to approximately 2.4×10^{20} cm^{-3}. Using this value of charge density in Poisson's equation, the maximum length of film which could be doped to this level without compensating anionic charges is approximately 1.7×10^{-7} cm, 5 orders of magnitude smaller than the length of the device (L = 5×10^{-2} cm). Thus, we can assume that the induced holes measured optically in the Type I doping front are compensated with equal and opposite ionic charge.

Supplementary Note 3: Internal electric fields during Type I and Type II doping.

During doping and dedoping front experiments, the internal electric fields across the material will evolve with time due to the different charge carrying species. In this section, we discuss how the optical data can be used to infer the internal fields.

First, we discuss the assumptions made for this analysis. The first assumption we make is that ionic carriers can cross the polymer-electrolyte interface (left side) freely and cannot cross the polymer-ITO interface (right side). Conversely, electronic carriers can freely cross the polymer-ITO interface and cannot cross the polymer-electrolyte interface. Second, we assume that changes in $\Delta T/T_0$ are electroneutral at the length scale probed by our optical measurements as discussed in **Supplementary Note 2**. Thus, wherever the derivative d*T*/d*t* peaks is where the ion and hole currents converge in space. This assumption also results in a constant current density along the length of the polymer. Finally, we assume that charge neutrality can be broken at the Debye length which will result in nonlinear potential drops along the length of the film according to the electrostatic 1D Poisson's equation (**Equation S7**).

$$\frac{\partial^2 V(x)}{\partial x^2} = \left(C_{anion}(x) - p(x) - C_{cation}(x)\right)\frac{e}{\epsilon_0 \epsilon_r} \qquad \text{(Equation S7)}$$

V and *x* are the potential and distance along the length of the polymer, respectively, C_{anion}, C_{cation}, and p are the concentrations of anions, cations, and holes along the length of the polymer, respectively, e is the elementary charge, and ϵ_0 and ϵ_r are the permittivity of free space and relative dielectric constant, respectively.

When the potential is initially applied to the ITO contact and before charges move, from **Equation S7** we would expect a linear potential drop along the length of the polymer since the $\frac{\partial^2 V(x)}{\partial x^2}$ must be 0. Then, as charges drift, the electric field will evolve such that most of the potential drop is across the highest resistance element in the circuit following Ohm's law. For doping and dedoping of PEDOT:PSS, holes are highly mobile and thus the conductivity of holes is higher than that of ions, so the potential drop occurs primarily across the ionic part of the circuit resulting in an initial peak in the derivative at that interface (**Fig. 2b**). The potential of the electrolyte is fixed so the potential flattens over time resulting in diffuse doping/dedoping characteristics (**Fig. 2a**)

Unlike doping/dedoping in PEDOT:PSS, during doping of p(g1T2-g5T2), d*T*/d*t* initially peaks near the ITO contact. Thus, we can infer that the initial ion drift current must exceed the initial hole drift current. Rather than broadening with time, the peak originating near the ITO contact moves across the length of the film linearly and the intensity remains nearly constant. This result indicates that, with time, the convergence point and of J_{ion} and J_{h} moves towards the electrolyte as doping proceeds, but the doping rate (d*T*/d*t*) is invariant with time and as the length of the moderately doped (poorly doped) region increases (decreases). That is, J_{ion} is equal to J_{h} and both remain constant with time. If the rate of doping were limited by transport of ions from the electrolyte to the region of doping, the rate of doping would accelerate as the distance ions must travel decreases. Similarly, if the doping rate were limited by hole transport through the moderately doped region, the doping rate to slow down as the distance holes must travel increases. Thus, we can conclude that the process limiting the speed of doping occurs at the boundary between the moderately doped and the poorly doped parts of the film, and the drop in potential should be largest at this rate-limiting boundary.

From our characterization of μ_h as a function of potential (and therefore doping level), we know there is a sharp transition in μ_h between carrier concentrations of approximately 1×10^{20} cm^{-3} (-0.2 V *vs.* Ag/AgCl) and 2.4×10^{20} cm^{-3} (0.0 V *vs.* Ag/AgCl). The doping level reached during Type I doping is within this sharp μ_h transition. Therefore, we attribute the transport of holes from the doped (conductive) region of the film into the poorly doped (nonconductive region) as the rate limiting step. We also note that once the entire polymer film is moderately doped (when the Type I doping front reaches the polymer-electrolyte interface), the rate of doping increases substantially as observed by the peak in dT/dt at approximately 3 s (**Fig. 2f**).

The results from turning on of OECTs are also consistent with the explanation above (**Fig. 4**). During the hole-limited doping regime, the gate-source current is constant with time, consistent with doping being limited at the interface between the moderately doped and poorly doped regions of the polymer (**Fig. 4f**). Additionally, the doping front moves away from the source and drain towards the centre of the channel linearly with time (**Fig. 4d**). The measured potential drops along an OECT channel confirm a delay in the rise of the potential measured further away from the source electrode (**Extended Data Fig. 8**), indicating that the region of largest potential drop follows the propagation of the doping front from the source electrode towards the drain electrode (**Fig. 4c** and **Fig. 4d**).

To better understand the driving forces for ion and hole motion during doping front experiments, we measured the internal potential drops along the length of p(g1T2-g5T2) channel (L = 2.5 mm, W = 0.4 mm) using inserted voltage probes (0.5 mm spacing) along the channel as schematically depicted in **Extended Data Fig. 3a** (note that the gate is not drawn to scale). Briefly, the sample was prepared by making OECTs with inserted voltage probes (30 µm width) using the same methodology described in the methods section followed by spin coating of polystyrene from a 20 mg/mL solution in toluene. Then, a sharp tweezer was used to scratch a trench for exposing the film to the electrolyte at the position of the 5th voltage probe, approximately 2.5 mm from the gold contact. Potentials were measured at each probe (V_{1-4}) using a 4-channel oscilloscope (Keysight DSOX1204G) while a source-measure unit (Keysight B2902A) was used to apply a pulse between the Ag/AgCl gate and the gold contact. We note that the voltage probes measure the electrochemical potential of holes with respect to the grounded gold contact at the end of the channel, which includes contributions due to the local electric potential as well as shifts in the Fermi level resulting from doping. The resulting internal potential profiles in the hole-limited doping regime show that initially, the entire channel rapidly reaches the potential of the electrolyte, indicating a voltage drop primarily between the hole injecting gold contact (which is grounded) and the polymer (**Extended Data Fig. 3b**). As the Type I doping front proceeds, the potential at each probe increases starting with the probe nearest the hole injecting gold contact (V_4) to the probe nearest the electrolyte (V_1), indicating that the region of largest potential drop moves from the grounded gold contact and propagates towards the electrolyte. This result is highlighted by the darker colour traces. In contrast, during Type II doping (V_G from -0.3 V to -0.6 V), the internal potential of the entire channel drops rapidly to the change in electrolyte potential (-0.3 V) (**Extended Data Fig. 3c**). Then, the potential at all of the probes increases simultaneously while maintaining a linear profile, leaving the largest potential drop between the electrolyte and channel. This is also observed in the time dependent voltage measurements at each probe (**Extended Data Fig. 3d-g**) (time = 0 ms denotes the time when a potential is applied), where the onset of potential increase is delayed from the time of applied voltage only in the hole-limited doping regime (**Extended Data Fig. 3f**). The timescales of this experiment vary from the optical experiments, likely due to substantial differences in sample preparation.

Supplementary Note 4: Generality of hole-limited doping in conjugated polymers

The heterogeneous disorder found in p(g1T2-g5T2) is likely common among mixed conducting polymers. Thus, we expect other conjugated polymer electrodes to exhibit a hole-limited doping regime at sufficiently low doping densities and timescales. To test this hypothesis, doping fronts were measured for p(g0T2-g6T2), which has an identical thiophene backbone as p(g1T2-g5T2) with a different distribution of ethylene glycol sidechains, p(g2T-TT), a commonly reported p-type OMIEC material,[1] as well as for PEDOT:PSS (**Extended Data Fig. 4**). All of the polymers tested show a steep drop in hole mobility and volumetric capacitance at reducing potentials in OECTs (**Extended Data Fig. 5**).

The doping front in p(g0T2-g6T2) ($V_{WE,i}$ = -0.4 V *vs.* Ag/AgCl, $V_{WE,f}$ = 0.6 V *vs.* Ag/AgCl) travels in the contact-to-electrolyte direction, indicative of hole-limited doping, but the front originates from the centre of the film (**Extended Data Fig. 4a**). This result suggests that, at the initial doping state, ion and hole drift conductivities in p(g0T2-g6T2) are nearly equal, leading to doping in the centre of the film. The location of rapid doping propagates towards the electrolyte (I) like doping of p(g1T2-g5T2) but at a faster rate. Once the front reaches the electrolyte, doping proceeds via ion-limited doping (II). Lowering the initial doping state of p(g0T2-g6T2) ($V_{WE,i}$ = -0.6 V vs. Ag/AgCl) causes the initiation of the doping front to shift to the ITO contact (**Supplementary Figure 2**), closely resembling doping in p(g1T2-g5T2).

Testing doping of PEDOT:PSS from the poorly doped state requires relatively large voltages ($V_{WE,i}$ = -1 V *vs.* Ag/AgCl) to ensure that PEDOT:PSS is fully dedoped. When sufficiently dedoped, the subsequent doping shows localisation at the ITO contact, indicative of hole-limited doping (**Extended Data Fig. 4b**). The hole-limited doping occurs synchronously with a forward propagating doping front from the electrolyte/polymer interface, which likely results from soluble O_2 chemically oxidizing highly reduced PEDOT:PSS.[2,3] Nevertheless, the presence of a Type I doping front near the ITO contact, which is far from the dissolved O_2, indicates that a hole-limited doping regime exists even for PEDOT:PSS.

PEDOT:PSS and p(g0T2-g6T2) both exhibit transitions from hole-limited (I) to ion-limited doping (II) with a corresponding shift in the differential transmission spectra (**Extended Data Fig. 4d, 4e**), indicating that the transition from hole-limited to ion-limited doping at low hole concentrations is caused by heterogeneous disorder. The much smaller effect of hole-limited doping in p(g0T2-g6T2) and PEDOT:PSS are attributed to the lower degree of spatial disorder and lower degree of heterogeneous energetic disorder for pg0T2-g6T2 and PEDOT:PSS,[4] respectively.

The doping of p(g2T-TT) ($V_{WE,i}$ = -0.3 V *vs.* Ag/AgCl, $V_{WE,f}$ = 0.6 V *vs.* Ag/AgCl) also initiates at the ITO contact and moves from the contact to the electrolyte (**Extended Data Fig. 4c**). However, rather than showing a linear dependence between the time and distance, the doping front appears to slow as it approaches the electrolyte-CP interface, approximately following a doping time proportional to the square of the distance. This result indicates that the moderately doped region of the film continues to have a high resistance to hole transport, and the resistance for doping increases as the length holes must travel from the contact increases, similar to previously reported two resistor models.[5] In other words, holes continue to limit the speed of doping even as the polymer becomes moderately doped. We find a similar distance squared dependence for the OECT turn-on delays in p(g2T-TT) OECTs with increasing channel lengths (**Supplementary Figure 6**)

There is no sharp transition from hole-limited to ion-limited doping which may be due to the lower mobilities and more gradual increase in capacitance *vs.* voltage compared to p(g1T2-g5T2) and p(g0T2-g6T2) (**Extended Data Fig. 5**). The changes in the differential transmission spectra are also minimal (**Extended Data Fig. 4f**), indicating that the hole-limited doing in p(g2T-TT) may not be directly related to different types of ordering in the CP microstructure.

Table S1: Ionic drift-diffusion simulation parameters

Simulation parameter	Value [units]
Distance step (dx)	2.5×10^{-4} [cm]
Time step (dt)	1×10^{-5} [s]
Sulfonate anion concentration (N_0)	2.08615×10^{21} [cm^{-3}]
Length (L)	5×10^{-2} [cm]
Cation mobility (μ_{cat})	3.5×10^{-5} [cm^2 V^{-1} s^{-1}]
PEDOT:PSS volumetric capacitance (C^*)	40 [F cm^{-3}]
Initial hole concentration (p_0)	2.45515×10^{20} [cm^{-3}]
Initial cation concentration (C_0)	1.840635×10^{21} [cm^{-3}]

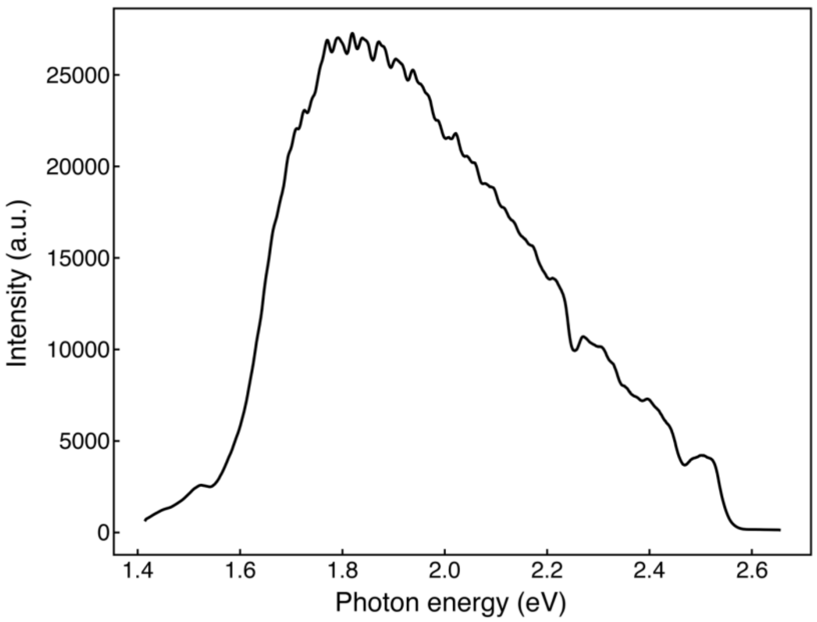

Supplementary Figure 1. Transmitted lamp spectrum. Relative spectral intensity of transmitted light in the transmission microscope.

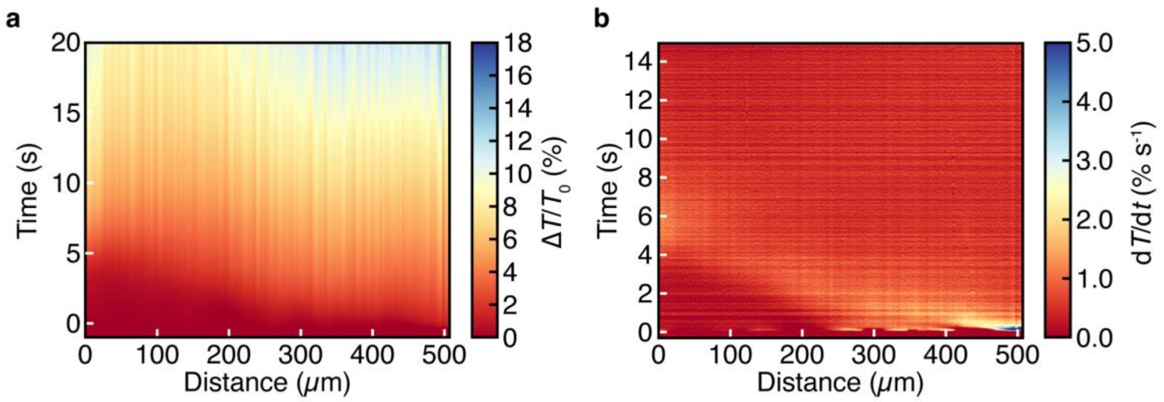

Supplementary Figure 2. Electrochemical doping of p(g0T2-g6T2) from -0.6 V *vs.* Ag/AgCl. Spatiotemporal transmission heatmap **a**, and corresponding time derivative **b**, for the doping transition of p(g0T2-g6T2) from -0.6 V to 0.6 V *vs.* Ag/AgCl in the moving front device geometry.

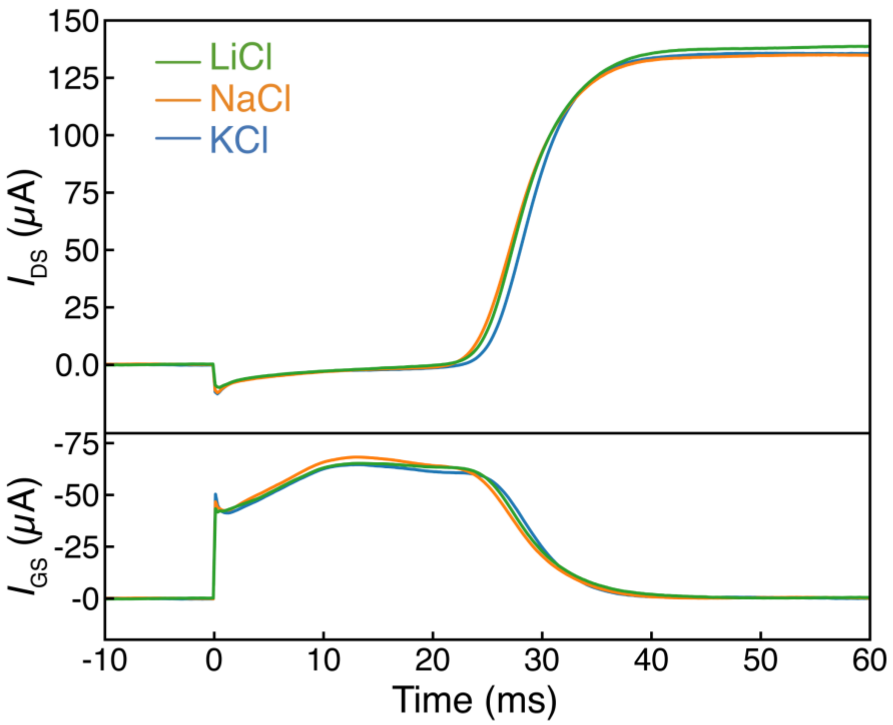

Supplementary Figure 3. Hole-limited doping with varied cationic species. Turn-on transients for p(g1T2-g5T2) OECTs (*L* = 800 µm, *W* = 100 µm) using a series of cationic species (0.1 M in water) with varied affinity to ethylene glycol sidechains.

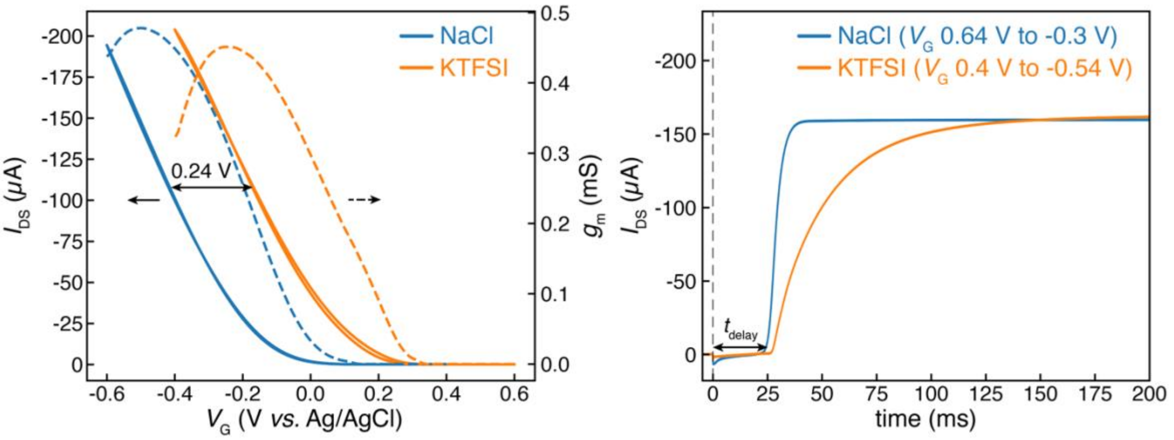

Supplementary Figure 4. Hole-limited doping with varied anionic species. a, Transfer characteristics and **b**, turn-on transients for p(g1T2-g5T2) OECTs (L = 400 µm, W = 100 µm) using aqueous solutions of sodium chloride (0.05 M NaCl) and potassium bis(trifluoromethylsulfonyl)imide (0.02 M KFSI). The initial and final gate potentials for measuring turn-on transients are adjusted due to the offset in potential (0.24 V) relative to the Ag/AgCl pseudo-reference gate electrode caused by switching the anion from Cl^- to $TFSI^-$. The delay time due to hole limited doping is comparable for both anions while the switching speed of the OECT operated in aqueous KTFSI is much slower.

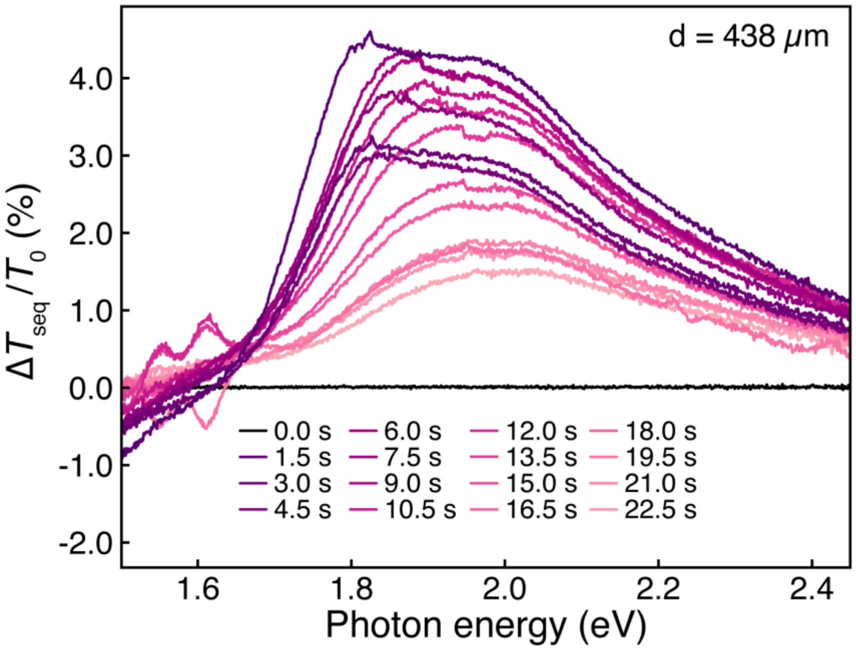

Supplementary Figure 5. Sequential differential transmission spectra during doping of p(g1T2-g5T2) before normalisation.

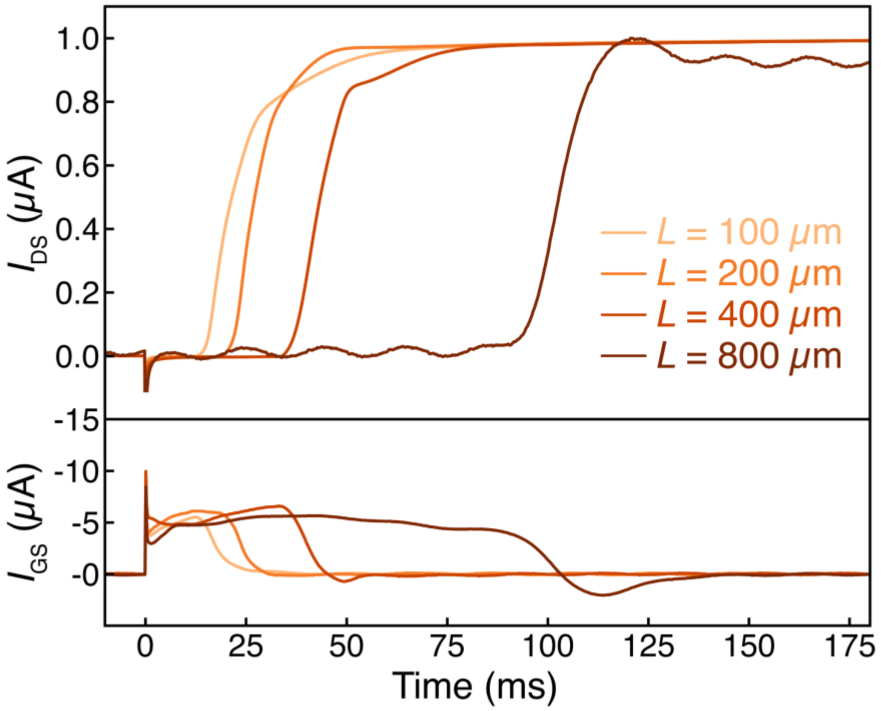

Supplementary Figure 6. Length dependence for switching time transients in p(g2T-TT) OECTs. Unlike p(g1T2-g5T2) where the switching time delay caused by hole-limited doping increases roughly linearly with the channel length (**Fig. 4f**), the switching time delays in p(g2T-TT) OECTs appear to increase with the distance squared (W = 100 μm).

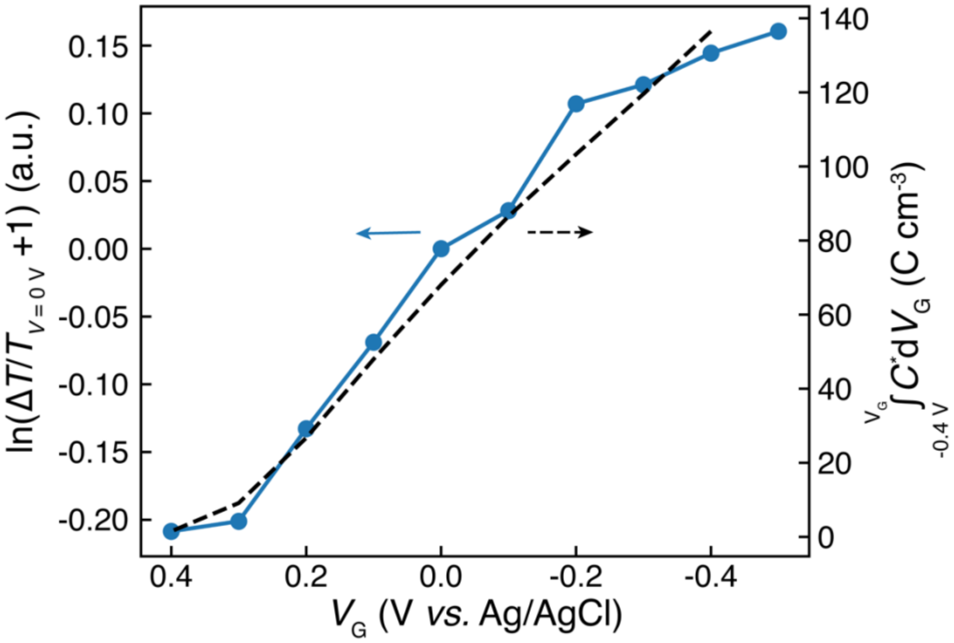

Supplementary Figure 7. Relationship between the applied potential and transmitted intensity. The natural log of the change in transmitted intensity ($\ln[\Delta T/T_{V=0\,V} + 1]$) with respect to the transmission at $V_G = 0$ V *vs.* Ag/AgCl for a transparent OECT averaged over the entire device area plotted as a function of the applied gate potential (V_G) (blue curve, left y-axis). From **Equation S6**, we expect $\ln(\Delta T/T_{V=0\,V} + 1)$ to be proportional to the hole concentration. We compare the result to the relative hole concentration, estimated as the integral of volumetric capacitance (C^*) *vs.* V_G measured using OECTs. Briefly, the carrier concentrations in the OECT are estimated using the volumetric capacitance given by **Equation S8** below.

$$p = V * C^* \qquad \textbf{(Equation S8)}$$

Where C^* is the volumetric capacitance of the material. Thus, the change in carrier density (Δp) is proportional to the integral of the voltage dependent C^* as follows in **Equation S9**.

$$\Delta p = \int_{V_i}^{V_f} C^*(V)dV \qquad \textbf{(Equation S9)}$$

Where V_i and V_f are the initial and final applied potentials, respectively. Finally, we can verify insert the estimation for p based on C^* into **Equation S6** to get the following proportionality in **Equation S11**.

$$ln\left(\frac{\Delta T}{T_0} + 1\right) \propto \int_{V_i}^{V_f} C^*(V)dV \qquad \textbf{(Equation S11)}$$

The plot in **Supplementary Figure 7** illustrates the strong correlations among the potential, the concentration of holes, and the transmission intensity.

References

1 Giovannitti, A. *et al.* Controlling the mode of operation of organic transistors through side-chain engineering. *Proceedings of the National Academy of Sciences* **113**, 12017-12022, doi:10.1073/pnas.1608780113 (2016).

2 Giovannitti, A. *et al.* Energetic Control of Redox-Active Polymers toward Safe Organic Bioelectronic Materials. *Advanced Materials* **32**, 1908047, doi:10.1002/adma.201908047 (2020).

3 Keene, S. T., Melianas, A., Van De Burgt, Y. & Salleo, A. Mechanisms for Enhanced State Retention and Stability in Redox-Gated Organic Neuromorphic Devices. *Advanced Electronic Materials* **5**, 1800686, doi:10.1002/aelm.201800686 (2019).

4 Keene, S. T. *et al.* Efficient Electronic Tunneling Governs Transport in Conducting Polymer-Insulator Blends. *Journal of the American Chemical Society*, doi:10.1021/jacs.2c02139 (2022).

5 Stavrinidou, E. *et al.* Direct Measurement of Ion Mobility in a Conducting Polymer. *Advanced Materials* **25**, 4488-4493, doi:10.1002/adma.201301240 (2013).
